# Report on the Present State of Our Knowledge of the Nature of Inflammation

**Published:** 1844-04

**Authors:** T. Wharton Jones

**Affiliations:** Lecturer on Anatomy, Physiology, and Pathology at the Charing-Cross Hospital; Corresponding Member of the Imperial and Royal Society of Physicians and Surgeons at Vienna, &c. &c.


### REPORT ON THE PRESENT STATE OF KNOWLEDGE OF THE NATURE OF INFLAMMATION.

By T. Wharton Jones, F.R.S.

Lecturer on Anatomy, Physiology, and Pathology at the Charing-Cross Hospital; Corresponding Member of the Imperial and Royal Society of Physicians and Surgeons of Vienna, &c. &c.

When a part of the body, visible externally, is the seat of inflammation, the observer perceives it to be preternaturally red, swollen, and preternaturally hot; the patient, moreover, says that he feels it hot and painful. The conditions on which these symptoms depend are, on the whole, sufficiently obvious; but the nature of the process which leads to them—in other words THE THEORY OF INFLAMMATION—is very obscure, and has always been, and still is, an object of much debate and inquiry with pathologists.

As it is microscopical parts which are immediately concerned in the inflammatory process, it is only since pathologists became acquainted, by means of the microscope, with those parts and their mode of action, that any real advance has been made in the inquiry. The parts referred to are principally the corpuscles of the blood and the capillary vessels. In entering, therefore, on the task of reporting on the theory of inflammation, it will be useful to premise the following points regarding the blood and its circulation in the capillaries in the healthy state:

### I. BLOOD IN THE HEALTHY STATE.

§ 1. In *human blood,* after being drawn, the red corpuscles aggregate together like coins in rolls. This aggregation takes place in the course of half a minute or so after the blood is drawn, when the blood is in a healthy state, with its plasma of ordinary thickness; when, on the contrary, the blood is not in the healthy state, but has a more inspissated plasma, the aggregation of the red corpuscles is so rapid that it is found to have already taken place by the time the blood can be transferred to the microscope and examined.

§ 2. The admixture of certain reagents with the blood influences the aggregation. Some promote it, others prevent it; those which promote it, if anything, give rise to endosmose rather than to exosmose; among those which prevent it are found as well such as give rise to exosmose as those which give rise to endosmose. It appears to be viscidity of the fluid (a certain proportion of salts being at the same time dissolved in it,) in which they are suspended, which especially promotes, though is not the cause of, the tendency to the aggregation of the red corpuscles.

§ 3. In the newly-drawn *blood of the frog* a sort of aggregation of the red corpuscles is also observed; but instead of having their surfaces fully applied to each other, and being raised up on edge, as in human blood, the red corpuscles, for the most part, lie flat, and merely partially overlap each other, like the coins of a roll which has been thrown loosely down. The admixture of mucilage of gum with a little common salt in solution, which, in human blood, much augments though in an irregular and confused manner, the aggregation of the red corpuscles, has comparatively little effect on the blood of the frog. Henle states that white of egg causes the red corpuscles of frog's blood to aggregate into heaps; but this does not appear to be to any greater amount than when mucilage of gum, with a little salt in solution, is the reagent employed.

§ 4. Though the red corpuscles of the blood of the frog have less disposition to aggregate than those of human blood, the colourless corpuscles appear to have more.

§ 5. The red corpuscles of the blood of the frog, as seen circulating within the vessels of the web, do not show the nucleus, or that very indistinctly; but when examined after being drawn from the body, the presence of the nucleus is sufficiently evident. The immediate cause of this difference appears to be, that in the former case the red corpuscles are more distended, in the latter more collapsed. For the reason that they are more collapsed after being drawn, the red corpuscles also appear redder. This change in the red corpuscles is coincident with the occurrence of the tendency to aggregate.

### II. Capillary Circulation in the Healthy State.

§ 6. As the capillary circulation cannot be observed in the human body, reference is made to observations on the web of the frog's foot for what is necessary to be said on the subject, as a preliminary to inquiries into the action of the blood and of the capillaries in inflammation. In pursuing these latter inquiries, recourse must also be still had to observations on the web of the frog's foot. And it was in reference to this necessity to have recourse to observations on the frog, that the above remarks regarding its blood in comparison with that of man were made.

§ 7. When the circulation in the web of the hind foot of a frog is carefully observed under the microscope, the colourless corpuscles of the blood are seen, especially if the velocity of the stream be diminished by pressure on the limb, for example, accumulated on the inner surface of the walls of the vessels, along which they slide or roll over and over very slowly in comparison with the red corpuscles which occupy the axis of the stream, and move directly onwards.

§ 8. From these differences in the position, and in the mode and rapidity of progression of the two kinds of corpuscles of the blood, it would appear that there exists some sort of attraction between the colourless corpuscles and the walls of the vessels, but an absence of attraction, if not a repulsion, between the red corpuscles and these walls, as also between the red and colourless corpuscles.

§ 9. Though the red corpuscles keep together in the axis of the stream, there is not apparent among them any tendency to aggregate, like what is observed when the blood is drawn from the body.

§ 10. Through the very smallest capillaries, besides plasma and a few colourless corpuscles one after the other, a red corpuscle is only now and then observed to pass—a circumstance which is to be explained, not by the too great size of the red corpuscles, (for they can readily accommodate themselves to vessels of a diameter less than their own,) but by a reference to the circumstance of an absence of attraction or the existence of a positive repulsion between the red corpuscles and the walls of the vessels. The plasma and colourless corpuscles, by virtue of their attraction for the walls of the vessels, readily enter very small ones, but when a red corpuscle would enter, it comes within the sphere of the repulsion of the walls of the vessels, and is as it were warded off. And when it does happen that a red corpuscle enters one of the smallest capillaries, it appears to be only by being actually forced in by accidental pressure from behind.

### III. Phenomena Attending the First Steps of the Inflammatory Process, Visible by the Microscope.

§ 11. To proceed now with the inquiry into the nature of the inflammatory process.

If the web of the frog's hind foot, displayed under the microscope, be irritated, mechanically or chemically, an opportunity is obtained of witnessing what is microscopically observable of the first steps of the traumatic inflammatory process which is excited.

§ 12. Very soon, then, after the web of the frog's foot, thus displayed under the microscope, has been mechanically or chemically irritated, accumulation and stagnation of the blood in the capillaries, including the terminations of the arteries and radicles of the veins of the part, is observed to take place; but amidst the obstructed vessels a few here and there may still be seen pervious, and through them the stream of blood is very rapid. The accumulation and stagnation of the blood in the small vessels is always preceded by a retardation of its flow—this retardation of the flow of blood, having or not having been preceded by the opposite condition of an accelerated flow.

§ 13. The acceleration, when it does occur, and the retardation of the flow of blood are coincident with changes in the width of the vessels—the former with constriction, the latter with dilatation. Omitting from further consideration the accelerated flow of blood, and the constricted state of the vessels with which it is coincident, as not constant, let the *behaviour of the blood during the retardation of its flow and at the time of its stagnation* be inquired into.

§ 14. *a. Colourless corpuscles.* During the retarded flow of blood immediately preceding stagnation, an accumulation of colourless corpuscles is observed to take place on the inner surface of the walls of the dilated small vessels, similar to what occurs in the healthy state when the velocity of the stream of blood is diminished. (§ 7.)

§ 15. *b. Red corpuscles.* While the accumulated colourless corpuscles may have even become stagnant on the walls of the vessels, the red corpuscles, though in increased quantity, in proportion to the plasma, still continue to float on, but more and more slowly, until complete stagnation ensues. They are somewhat more collapsed than natural; hence they appear redder, and their nucleus is less indistinctly seen—a change similar to what takes place in the red corpuscles of newly-drawn blood. (§ 5.)

§ 16. The red corpuscles appear to be the agents principally concerned in the stagnation of the blood. The mode in which this is seen to be brought about was, for the sake of contrast with the phenomena attending the circulation in the capillaries in the natural state, briefly described on a former occasion in this Review. (Oct. 1842.) It was there stated to be by the red corpuscles agglomerating together, and applying themselves here and there flat against the walls of the vessels, and adhering to them; whilst other red corpuscles applied themselves to those already adherent.

§ 17. The same phenomena had been described by others before; and Emmert* and Vogel,† in their publications of about the same date, have given similar but more detailed accounts of the mode in which the stagnation of the blood is seen to take place.

According to Emmert (ut supra, pp. 72-84,) the colour of the red corpuscles becomes somewhat deeper, whence they appear individually less transparent. The surface appears less smooth, the inequalities of the edges can be perceived with peculiar distinctness. The corpuscles at the same time acquire the property of remaining adherent to each other and to the walls of the vessels when they come into contact with them. If attention be directed to the streams of blood in the radicles of the veins which have just come out from the dilated capillary network, the adhesion of individual blood-corpuscles to each other, is still often to be seen. Sometimes they adhere to each other more with their points, so long as they lie behind each other; sometimes it is the lateral edges or some parts of the surfaces which are agglutinated.

Vogel's account is as follows: (pp. 315-26.)

When the flow of blood becomes retarded and oscillations commence, the blood-corpuscles apply themselves more to each other; the individual corpuscles may, indeed, be still perfectly distinguished from each other, but they touch each other, and are, in the smaller capillaries, often pressed close together by their surfaces, in the manner of rolls of coin. The space next the walls of the vessels appears to be merely filled with plasma. In complete stagnation of the blood, this space disappears, the interior of the vessel is completely filled with blood-corpuscles, which are closely aggregated together, and form an apparently homogeneous indistinctly granulous mass, in which individual blood-corpuscles can scarcely be distinguished. But this fusion is merely apparent; for if the blood be evacuated by opening the vessels, the individual corpuscles again appear quite distinctly.

§ 18. In reference to the account in this Review for October, 1842, above referred to, of the mode of stagnation of the blood in the capillaries of the web of the frog's foot, when irritated mechanically or chemically, Dr. Williams,‡ after noticing what the author had said of the aggregation of the red corpuscles of human blood, newly drawn during inflammation, observes, "But Mr. W. Jones is premature in assuming that a similar aggregation of the blood-corpuscles occurs within the blood-vessels, and is the cause of obstruction in the capillaries in inflammation and other cases of impeded circulation. No such cohesion is seen in

* Beiträge zur Pathologie und Therapie, Heft i; Bern, 1842.

† Wagner's Handwörterbuch der Physiologie, Art. 'Entzündung.' ‡ Principles of Med., p. 89, § 100.

the large vessels of a frog's web, when the motion of the blood is arrested by pressure on a vein; and although the blood does coagulate (?) in some of the vessels of an inflamed part, this will be hereafter shown to begin with the colourless rather than with the red particles."

§ 19. Leaving for after-consideration the latter proposition, the author of this Report would remark, that, considering the place and mode in which the above allegation is made, that he "assumes" that an aggregation of the blood-corpuscles similar to that presented by buffy blood occurs within the blood-vessels, and is the cause of obstruction in the capillaries in inflammation, one, confiding in the accuracy of Dr. Williams, would suppose that the author of this Report made the imputed assumption because the red corpuscles of human blood drawn from a person labouring under acute inflammation aggregate more rapidly and closely than in blood from a healthy person. How much soever, the author of this Report believes, as will be seen below, that the greatly increased tendency of the red corpuscles of buffy blood to aggregate would promote the action of the exciting cause of inflammatory stasis, it was never for a moment his opinion that such increased tendency was a necessary condition for inflammatory stasis, knowing that this may arise from a slight injury, and when the mass of blood is still quite healthy.

§ 20. But suppose Dr. Williams did not mean to impute any such opinion, but merely intended to say that the author of this Report "assumed" that an aggregation of the blood-corpuscles occurs within the blood-vessels, similar to that which healthy blood newly drawn presents, and is the cause of obstruction in the capillaries in inflammation, the author begs to observe again, that how much soever he believes it, as will be seen below, he did not, in the paper referred to, either state it or assume it. He merely stated, without any particular reference to the aggregation of the red corpuscles of human blood, what he had *observed*, what others have observed, and what Dr. Williams himself appears to have *seen*, though he has not sufficiently reflected on it, in regard to the mode in which stasis takes place in the capillaries of the web of the frog's foot, in consequence of mechanical or chemical irritation, and contrasted it with the phenomena attending the circulation in the capillaries in the natural state. Further, if the author did not either state or assume any such thing in regard to inflammation, much less did he do so in regard to "other cases of impeded circulation." Had the author stated or assumed this, Dr. Williams would certainly have been correct in bringing forward, in refutation of it, the argument he does. But when Dr. Williams makes use of the argument referred to in refutation of the other "assumption" which he imputes to the author, viz., that aggregation of the red corpuscles within the blood-vessels is the cause of obstruction in inflammation, he errs very greatly. The condition of blood arrested in the capillaries by pressure on a vein is surely quite different from that of blood in the relaxed and dilated vessels at the time of inflammatory congestion, or of blood after it has been drawn from the body.

But to return from this digression:

§ 21. *c. Plasma.* This, it has been said, (§ 15,) becomes proportionally diminished in quantity in consequence of the accumulation of the red corpuscles. How far this proportional diminution in the quantity of plasma is owing to its draining away from among the stagnating red corpuscles into the veins, and how much to the serous exudation through the walls of the vessels which occurs about the time of stagnation, will form a subject of inquiry further on.

### IV. EXPLANATION OF THE MICROSCOPICALLY-VISIBLE PHENOMENA ATTENDING THE FIRST STEPS OF THE INFLAMMATORY PROCESS.

§ 22. Retardation of the flow of blood in the small vessels, and that coincident with dilatation of their caliber, and at last accumulation and stagnation of the blood-corpuscles in the vessels thus constitute the first phenomena constantly appreciable by the microscope in the inflammatory process, as observed in the frog. The *macroscopical* phenomena of inflammation in man seem to warrant the inference that the *microscopical* ones are essentially the same in him as in the frog. The explanation of these phenomena, therefore—their sequence and relations—is justly considered the key of the whole theory of inflammation.

§ 23. Is dilatation of the small vessels primary, and retardation of the flow of blood in them secondary, or is the contrary the case? That dilatation is primary and retardation of the flow of blood the necessary physical result of the preceding dilatation, is maintained by most recent authors. And though they have good reason on their side, it is to be observed that, considering the peculiar vital endowments of the blood and the vessels, the fact of the case is not so unconditional as it might seem at first to be, judging merely from the flow of non-vital fluids in inert tubes. For, as accumulation and, lastly, stagnation of the blood soon supervene on the retarded flow, and as that accumulation and stagnation must, as will be shown, acknowledge some other cause than dilatation of the vessels, this retarded flow might be attributed, as is done by Vogel, to the commencing operation of that cause, whatever it is, which determines the accumulation and stagnation; and the dilatation itself to distension from the accumulated blood, and therefore secondary. To this it is to be replied, however, that dilatation of the vessels from distension by accumulated blood would be subsequent, by an appreciable interval, to the retardation of the flow of blood, which it is not; for there is already dilatation with coincident retardation, before accumulation has taken place to an extent sufficient to produce distension.

§ 24. The opinion, therefore, above stated as that entertained by most authors is to be considered just, but only so far as it goes, for it does not embrace the whole truth. The truth appears to be this:—Dilatation is primary, but the retardation of the flow of blood is in part only the physical effect of it, being greater than the dilatation is sufficient physically to account for. The other cause in operation is the same as that which at last determines the accumulation and stagnation of the blood, as will be explained below. By the accumulation of the blood, however, there is a secondary dilatation of the vessels—one from distension, but which more particularly implicates the capillaries—perhaps is the sole dilatation of which the capillaries proper are the seat, as will immediately be shown. This view is much the same as that entertained by Vogel in regard to certain cases only, viz., those in which common congestion passes into inflammatory congestion. It being Vogel's opinion that it is in common congestion only that dilatation of the vessels is primary, and that in inflammatory congestion it is from distension alone, and altogether secondary. But there do not appear to be any grounds for this opinion.

§ 25. Having thus determined that there is primary dilatation of the vessels, the next subject of inquiry is the nature of the dilatation. Does dilatation depend on an active state of the walls of the vessels, or on a state of relaxation? The prejudice that inflammation is a state of increased action of *all* the parts concerned, which has led some (justly believing that constriction is the active state of the vessels) to maintain that the vessels are constricted in inflammation, has led others (knowing that the vessels are really dilated in inflammation) to maintain that their dilatation is an active state. This view was most strenuously maintained by the late Professor Macartney of Dublin, and more recently it has been advocated by Lotze. (Allg. Pathologie und Therapie als mecanische Naturwissenschaften, Leipzig, 1842, pp. 277-368.) Dr. Copland maintains that in sthenic inflammation the vessels are actively dilated, and that a larger column of blood circulates in them with increased velocity. In asthenic inflammation Dr. Copland admits the dilatation of the vessels to be owing to relaxation, and that the flow of blood in them is retarded or arrested. John Hunter, while he admitted relaxation of the muscular powers of the walls of the vessels, spoke of the dilatation of the vessels as if it were an active state, believing it to be to a greater extent than could be permitted without force by the elasticity of the vessels. This, however, was evidently a mistake on the part of Hunter, produced by clinging to the prejudice of increased action. Eisenmann (Haeser's Archiv, 1841, pp. 239-349), and Heine (Physio-pathologische Studien; Stuttgardt und Tübingen, 1842, p. 156), again justly believing that constriction is the active state of the vessels, and at the same time aware that in inflammation there is dilatation of vessels, have sought to reconcile this fact with the prejudice that inflammation is a state of increased action, by supposing, as Haller had done, the existence of spasmodic contraction in one part of the vessels and dilatation from distension immediately behind; Eisenmann believing the distended part of the vessel still in an active state, but its contractile force overcome by the distension from within; Heine again supposing that the distended part is relaxed like the part of intestine behind the contracting part.

Farther details of these and such views it is unnecessary here to give, as the arguments brought forward in their support are too fallacious—too inconsistent with established physiological principles, or as they are opposed to the results of direct observation.

§ 26. Animal physiology recognizes no other motor agent than contractile fibre, i. e., a fibre capable, under certain conditions, of becoming shortened in the direction of its length, and that with force, but when no longer under these conditions readily resuming its former length. As therefore the walls of vessels are formed of circular contractile fibres, diminution or constriction of caliber must be the only result of their active state, and dilatation of their caliber the result of their relaxation or passive state.

§ 27. To this add the results of the direct observation among others, of Alison:—" In a series of observations made in Edinburgh," says Alison (Outlines of Pathology and Practice of Medicine, pp. 116-117), " on the arteries leading to inflamed limbs in horses at different parts of their course, and at different periods of the inflammation, it always appeared that these vessels possessed less of the only vital power which experiments authorize us to ascribe to them; that they had less power to propel their contents than those of the opposite sound limbs.*

§ 28. As to the small arteries, the dilatation of their caliber, as observed taking place in the web of the frog's foot under the microscope, one would think could suggest no other interpretation than that it is an analogous state of relaxation to what is more unequivocally appreciable in the larger arteries, whilst diminution of their caliber is a state of activity of their walls. But, according to Lotze, it is a prejudice to admit, from analogy, that the walls of the fine vessels must contract like the larger in the state of activity. He is of opinion that it is not unscientific to view in the one a condensation, in the other a separation of the molecules, as the consequence of nervous activity. Henle, in objecting to this, well remarks, " Whoever has himself made researches in any department of natural science, will not estimate lightly proof from analogy. Error is indeed possible, and a conclusion from analogy therefore remains an hypothesis until observation has confirmed it. But it is somewhat different to distrust such an hypothesis, or as Lotze does, to set up an hypothesis which is directly opposed to analogy." ('Bericht,' p. 97.)

§ 29. It is to be concluded then, as first suggested by Vacca, and in corroboration of which microscopical observations were first adduced by Wilson Philip, and now admitted by most authors on the subject, that the dilatation of the arteries in inflammation is a state of relaxation or paralysis, not of activity. Having come to this conclusion the next inquiry is as to whether the capillaries and venous radicles have contractile coats, and are therefore subject to dilatation from relaxation.

§ 30. Though constriction and dilatation of the capillaries and radicles of the veins are said to take place as well as constriction and dilatation of the small arteries, it is proper to observe that it is the latter alone which are seen under the microscope to be the seat of such marked constriction and dilatation of their caliber, as appear to be owing to an action of their coats of the same nature as vital contractility. So marked is the difference in this respect between the small arteries and the capillaries, that whilst the caliber of the artery may be observed to become almost wholly obliterated for the time by contraction of its walls throughout the whole

* The increased force with which the arteries leading to an inflamed part throb, thus cannot be owing to increased action. It is owing, as pointed out by Dr. Billing and Mr. Davies, to this, that, being relaxed, they yield more readily to the impulse of the blood propelled into them at each stroke of the heart.

extent of the part of the vessel under observation, or at intervals presenting the appearance of a series of strictures, a varicose appearance, as Wedemeyer expresses it, the capillaries into which it opens continue to preserve their caliber little or not at all changed.

§ 31. It is to be remarked, in opposition to this, that Emmert, who formerly denied the contractility of the walls of the capillaries, has recently admitted it, and estimated the diminution of their caliber by the contraction which, from moderate irritation, always precedes dilatation, to amount to one quarter or one fifth of the diameter of the vessels. This amount of constriction of the capillaries is very small in comparison with that presented by the arteries. The dilatation, again, of the capillaries succeeding the constriction, is stated by Emmert to be as much as one third to one half and more. The radicles of the veins do not present any greater diminution of caliber than the capillaries, though, like them, they admit of great dilatation.

§ 32. Not being satisfied that the capillaries and radicles of the veins have contractile walls, and admit, therefore, of primary dilatation from relaxation, the author of this Report is disposed to believe that dilatation of the capillaries and radicles of the veins is secondary to the retardation of the flow of blood in the arteries, and is owing to distension from the accumulating blood. The constriction of caliber which the capillaries are said to present, though to a small amount, may be ascribed to elastic reaction of their walls, as it exists at the time when the arteries are constricted and when the flow of blood is accelerated and not impeded by any tendency of the red corpuscles to accumulate. It is left undetermined whether rarefaction or condensation of the parenchyma in which the capillaries are distributed have any influence, as Vogel thinks, on their constriction or dilatation.

§ 33. It being thus certain, to use the words of Dr. Alison,* that during the whole time when inflammation and effusion consequent on it are most evidently going on, the condition of all the vessels (possessing contractile walls) leading to and passing through the inflamed part, is one not of contraction but of relaxation; the question before us, viz., whether the phenomena of inflammation can be explained by the alteration of the *vital powers of the vessels* in which the blood moves, is, Dr. Alison thinks, narrowed to this :– Does that state of relaxation afford a sufficient explanation of the changes which take place in the inflamed parts?

§ 34. The effect of relaxation of the vessels is dilatation, and the effect of dilatation is retardation of the flow of blood; though, as has been said, and as will be shown below, the whole amount of the retardation which takes place is not alone the direct physical effect of the dilatation. But putting this question aside for the present, let it be inquired what effect the retardation of the flow of blood has in producing accumulation and stagnation of the corpuscles.

1st. *Accumulation and stagnation of colourless corpuscles.*

§ 35. The colourless corpuscles which accumulate on the inner surface of the walls of the dilated vessels during the retardation of the flow of blood, have been alleged by Dr. Williams to be actually new formations occurring at the moment. Having directed attention particularly to this point, the author of this Report can venture to maintain that the colourless corpuscles which are observed to accumulate on the walls of the vessels are no new formations called forth at the moment; but that, as stated on a former occasion in this Review, they already exist in the blood. That when the velocity of the stream of blood is great, the colourless are mingled and carried along with the red corpuscles, but when the velocity of the stream is diminished from any cause—whether one of a temporary nature or that leading to inflammatory congestion—the colourless corpuscles become extricated from among the red ones and come into contact with the walls of the vessels, where, rolling slowly along or actually remaining stagnant, they accumulate in great numbers.

* Ut supra, p. 115.

The same view of the matter is taken by Emmert,*

2d. *Accumulation and stagnation of red corpuscles.*

§ 36. Has the retardation of the flow of blood any influence in determining the accumulation and stagnation of the red corpuscles? In other words, does retardation of the flow of blood lead to accumulation and stagnation?

The mere retardation of the flow of blood does not, as in the case of the colourless corpuscles, operate in determining the accumulation and stagnation of the red corpuscles. Whilst the colourless corpuscles always accumulate and stagnate, when from any cause retardation of the flow of blood takes place, (being, on account of their want of attraction for the red ones, extricated from among them, and by virtue of their attraction for the wall of the vessels brought into contact with it,) the red corpuscles having, under ordinary circumstances, no such attraction for the walls of the vessels, pass on along with the plasma.

§ 37. Accumulation and stagnation of red corpuscles not being, as is the case with the colourless corpuscles, determined by a retardation of the flow of blood in the vessels, how are their accumulation and stagnation determined? Has the accumulation of colourless corpuscles itself, which results from retardation of the flow of blood, any share in determining the accumulation and stagnation of the red corpuscles?

Dr. Williams conceives that the accumulation of colourless corpuscles, or, as he expresses it, the "increased production" of colourless corpuscles and their remarkable disposition to adhere to the walls of the vessels and one another, to be the chief cause of obstruction of the circulation in an inflamed part, and that by entangling the red corpuscles among them.

§ 38. This opinion is not more warranted by what is really to be observed by means of the microscope than is the view that the accumulation of colourless corpuscles is owing to an instantaneous increased production of them. At the time of stagnation of the red corpuscles there may be not more colourless ones than may often be seen at a time when the circulation is going on without any tendency of the blood to stagnate. Dr. Williams's own figure, p. 213, shows this. Besides, Dr. Williams has observed how the red corpuscles can accommodate themselves in order to glide past obstructions,—a circumstance which shows how little likely they are, when *otherwise unaffected by any change*, to be entangled by the colourless corpuscles or any other impediment not presenting a complete barrier.

§ 39. This explanation given by Dr. Williams, of the immediate cause of stagnation of the blood in the small vessels of an inflamed part, is a mere mechanical one; and though of a more refined character than most other such explanations, it is not in reality better founded. For a very able refutation of any explanation of the phenomena of inflammation on simply mechanical or chemical principles, or by any combination of the two, reference may be made to Alison's 'Outlines of Pathology and Practice of Medicine,' p. 108; and for a refutation of the latest explanation of the kind which has appeared on the continent, viz., that of Dubois, reference may be made to Henle's Report.†

§ 40. Do relaxation and dilatation of the vessels, with retardation of the flow of blood, considered by themselves, act in any way in determining stagnation of the blood?

Henle‡ thinks they do and that in the following manner:

As a physical consequence of dilatation of the vessels there takes place a retarded flow of blood. This retarded flow of blood, together with the relaxation and dilatation of the vessels, favours the exudation of serum; the consequence of which is, that the plasma of the blood in the part becomes inspissated by a preponderance of protein matter over the salts. This inspissation of the plasma determines endosmotic changes in the red corpuscles, in consequence of which they are disposed to aggregate.

§ 41. Henle, at first, gave this explanation of the immediate cause of stagnation

* Ut supra, p. 48.

† Bericht über die Arbeiten im Gebiet der rationellen Pathologie seit Anfang des Jahres 1839. p. 45.

‡ Ut supra, p. 130.

of the blood as *possible* only; but he now thinks that the appearances of the red corpuscles at the time of stagnation (§§ 5-15-17) being such as indicate the action of an inspissated plasma, render the opinion more probable.

§ 42. The author of this Report cannot agree with Henle, that relaxation and dilatation of the vessels are the first step to stagnation, merely by virtue of their allowing a retardation of the flow of blood in the affected vessels and a copious exudation of serum from them, so that the plasma becomes inspissated,—inspissation of the plasma being, as just stated, the condition which, according to Henle, immediately determines the agglomeration of the slowly flowing corpuscles and their subsequent stagnation.

§ 43. In the first place it may be doubted whether the serous exudation which accompanies inflammatory congestion, does not really follow instead of precede the stagnation;—certainly determination of blood has already taken place before exudation; and determination, it is to be remarked, is not owing to mere retardation of the flow of blood but also to accumulation of the red corpuscles, from what cause will be seen below. As to the rapid diminution in the quantity of the plasma, observed in the frog at the time of stagnation, it can be more easily supposed to be owing, as above hinted, §17, to its draining off from among the red corpuscles already beginning to aggregate than that the serous part of the plasma should have exuded in such quantity and so suddenly through the walls of the small vessels as to affect the blood flowing through them.

§ 44. The circumstance, otherwise very interesting, deduced by Henle from the analyses of blood drawn in inflammation, published by Andral and Gavarret, and by Simon, viz., that the chemical composition of the exuded serous fluid and of the blood in inflammation, stands in such a reciprocal relation as to show that the change in the blood might be in a great measure owing to the abstraction of the exuded fluid, does not prove that the serous fluid was exuded before the stagnation. And this, because in all the cases the blood analysed must have been drawn in the fully developed stage of the inflammation, and, of course, after stagnation had taken place, and when exudation might well have been subsequent to it.

§ 45. That the change which the blood drawn in the course of inflammation presents, however, is not owing to the mere abstraction of serum, is shown by this, that the red corpuscles are proportionally diminished in quantity instead of being increased as they ought to be, according to Henle's supposition, as to the cause of inspissation of the plasma. It is this diminution of the quantity of the red corpuscles which, according to the author of this Report, accounts for the preponderance of fibrin in the plasma, the red corpuscles having become resolved into it.*

* See the author's observations on the blood in this Review for Oct. 1842. Here the author would beg the reader's indulgence while he makes a few remarks in regard to himself personally. The view that the red corpuscles may be considered as glandular cells first occurred to him independently, but finding, on a reference to the General Anatomy of Professor Henle of Zurich, that that excellent and accomplished physiologist had anticipated him, he immediately sank all pretension to the view. Farther, Dr. Willis, to whom the author of this report had communicated his views regarding the signification of the red corpuscles, finding in Wagner's Physiology, which he has so well translated, a statement to the effect that the red corpuscles might be presumed to bear the same relation to the plasma and its normal composition, as the cells of secreting glands do to the secreted fluids, made it known to the author just as his paper on the blood was about being printed off. This view of Wagner as well as the similar one of Henle the author inserted without any reference to the circumstance that the same view had independently occurred to him. The view, however, that the more peculiar object of the elaboration performed by the red corpuscles, is the conversion of albumen into fibrin, and that the augmentation of fibrin in the blood in inflammation is at the expense of the red corpuscles, they, in consequence of their increased secretory action, being more quickly and in greater quantity, resolved into fibrin than in health, not having been expressed either by Wagner or Henle, he allowed himself to enunciate it as if it were his own. And in this he believes he was justified. Dr. Carpenter, however, in a report in this Review for Jan. 1843, who, it is to be remarked, could have gained his knowledge of the subject only from the author's paper and Willis's translation of Wagner, attributes the view to Wagner and Henle, merely adding the name of the author of this report after theirs. And, as is the fate of statements which pass from one compiler to another, the "Wagner, Henle, and Wharton Jones" of Dr. Carpenter is curtailed by Dr. Williams to "Wagner, Henle, and others !" In regard to all

§ 46. As to the appearances presented by the red corpuscles at the time of aggregation mentioned at §§ 15-17, they are no proof of inspissation of the plasma. Similar appearances are presented by blood after being drawn from the body (§ 5); and when care has been taken to prevent inspissation,—nay when the fibrin has been removed, and the red corpuscles are suspended in the serum merely. Besides inspissation of the plasma by an increase of fibrin, at the same time that it promotes aggregation of the red corpuscles, has a tendency to produce *en*dosmotic rather than *ex*osmotic changes.

§ 47. But suppose serous exudation does occur before stagnation, and consequent inspissation of the plasma,—unless the whole exudation of serum took place at once, it is hard to conceive how the blood in a part, though flowing slowly in the capillaries, could be much affected by it. For the portion of blood, for example, from which serum is being given out will have passed on into the veins before inspissation of its plasma has taken place to a sufficient amount to determine aggregation of its red corpuscles, supposing aggregation to be brought about in this way, and will have been replaced by a new quantity from the arteries.

§ 48. In this way exudation of serum might continue to go on without being followed by aggregation of the red corpuscles, until the plasma of the whole blood in the body became inspissated to the due extent, and then the following difficulty would present itself:—

§ 49. If mere inspissation of the plasma, together with a retarded flow of the blood, were the sole condition for the aggregation of the red corpuscles, and consequent stagnation in the small vessels, why, it may be asked, does not stagnation take place in the small vessels of any part in which the flow of blood is retarded by a bandage or any other means, when, as in acute rheumatism for example, the plasma of the whole blood is much inspissated? And why even do the red corpuscles not aggregate within the small vessels in the healthy and natural state of the plasma, when the course of the blood through these vessels is retarded by any cause, seeing that in blood out of the body the red corpuscles aggregate as well when the plasma is of natural consistence as when it is much inspissated only not so rapidly, though still rapidly enough?

§ 50. Stagnation of the blood must thus acknowledge some other essential cause than inspissation of the plasma. In fact, inspissation of the plasma is not at all under any circumstances the essential condition of the aggregation of the red corpuscles, either without or within the small vessels. When it exists it can merely promote the operation of the essential cause when this is allowed to come into play. This is an important distinction. (§§ 5-15-17-60.)

§ 51. Dismissing from consideration then, as unsatisfactory, the preceding explanations of the stagnation of blood in inflammation, the inquiry presents itself as to *whether stagnation of the blood in inflammation may not be referred to alteration of* powers, influencing the condition and motion of the blood *in the living body, and that independent of any contractions of living solids.**

§ 52. Dr. Alison having narrowed the question as to "whether the phenomena of inflammation can be explained by alteration of the vital powers of the vessels in which the blood moves," down to this, viz., "Does the state of relaxation in which all the vessels are in inflammation afford a sufficient explanation of the changes which take place in inflamed parts?" concludes (p. 121) that "inflammation and its effects are inexplicable by any alteration of the contractile powers of the living solids concerned in it; and necessarily imply an alteration of vital properties, by which the constitution of the blood, its relations to the surrounding textures and its movements through them are determined, but which are quite this, the author of this report would observe—that perhaps Wagner and Henle would repudiate the view as Dr. Carpenter himself does; if not, when they claim it, the author of this Report will yield it to them; but till then, he protests against the award of Drs. Carpenter and Williams. On an early occasion, the author of this report will meet Dr. Carpenter's objections to the view in question, and inquire into that which Dr. Carpenter proposes to substitute for it.

* Alison, p. 115.

distinct from any contraction of living solids. That such living properties exist (Dr. Alison goes on to say), that they effect the changes taking place at *insensible distances* among the particles of the blood, and that they are altered in inflammation, will hardly be denied by any pathologist. That they are capable of affecting the *visible* motion of the blood will appear a rash assertion only to those who have not accustomed themselves to consider the evidence by which it is supported."

§ 53. The conclusion that Dr. Alison comes to is, that inflammation consists essentially in a local increase of a vital property of attraction existing among the particles of the blood, and between them and the surrounding textures, and with which other vital properties are connected, and simultaneously excited. That the proximate cause of inflammation, although affecting the constitution of the blood, does not reside in blood only, but primarily in the agency on the blood of the solids through which it passes in the capillary vessels, appears clearly from the limitation of the disease to a certain locality in the body, from the fact of its easy reproduction, for a long time or for life, in the vessels which have once been the seat of it.

§ 54. It has been above stated, that according to Vogel, it is only in common congestion that the dilatation of the capillaries is primary, and then it has for its sole effect a retarded flow of blood, and that within narrow limits, not at all a total stagnation. The dilatation of the capillaries in inflammation on the other hand, Vogel thinks is secondary, and owing to mere distension from the accumulated and stagnated blood. He admits, however, that common congestion may precede inflammatory congestion, in which case dilatation of the vessels from relaxation will precede their distension by accumulated and stagnated blood.

§ 55. The cause of the congestion and stagnation of the blood in inflammation, Vogel considers to be a vital attraction betwixt the blood and the parenchyma of a part. This attraction he thinks is exerted in most cases by the parenchyma of the affected part, to which it is communicated either immediately by the exciting cause, or mediately through the nervous system. In other cases, besides depending on a change in the vital force of the parenchyma, the increased attraction depends also on a vital change of the mass of blood.

§ 56. The circumstance that the red corpuscles, from occupying the middle only of the stream, approach the wall of the vessel and completely fill it, Vogel thinks has its natural explanation in the alleged increased attraction between the blood and parenchyma, by which the blood-corpuscles naturally approach the walls of the vessel. The exudation of serum, which always takes place at the same time, and afterwards of plasma, he thinks also contributes by allowing the corpuscles to come closer together, and to the walls of the vessel.

§ 57. The exudation which immediately follows stagnation, Vogel readily explains by the increased attraction. The plasma passes out through the walls of the vessels as through a filter, but the corpuscles are retained.

§ 58. Emmert, entertaining the correct opinion that mere relaxation and dilatation of the vessels cannot suffice in any way to explain stagnation, considers the positive observation of adhesion of the red corpuscles to the walls of the vessels as an indication of the operation of attraction; but, as Henle remarks, there is an attraction of the corpuscles for each other, which cannot be explained by a reciprocal attraction betwixt the blood and parenchyma.

§ 59. Emmert considers the attraction to belong to the corpuscles only, and not like Vogel, in any way to the plasma, which is merely pressed out. Nor does he, like Vogel, admit any difference between inflammation and common congestion, believing that in both cases there is, besides the increased attraction above noticed, dilatation of the vessels.

§ 60. The appearances attending the stagnation of the red corpuscles (described in § 16-17) are such as might be supposed to be the effect of a suspension of the conditions by which, in the natural state, the red corpuscles keep in the middle of the stream, neither adhering to the walls of the vessels nor to each other, and do not readily enter the smallest capillaries; the effect in fact of the establishment of an attraction between the red corpuscles on the one hand and the walls of the vessels on the other, as well as among the red corpuscles themselves, instead of the absence of attraction or the actual repulsion which naturally exists. But supposing all this —supposing that attraction does come into operation, the question remains, How is the attraction called forth ? or what are the conditions on which it immediately depends ? or even which attend it ?

§ 61. Alison does not enter into this question particularly. He thinks that it is through the intervention of the nervous system that cold applied externally excites inflammation of internal organs ; but whether in all cases the exciting cause of inflammation act through the nervous system, he leaves undetermined. Amongst other objections which he mentions might be urged against this, is the fact, to be more fully noticed below, that inflammation occurs in an organ the nerves of which have been cut. This, however, as will be immediately seen, is no proof that it is not through the nervous system that the exciting cause of inflammation acts, for it may be not the presence but the absence of nervous influence which is the necessary condition.

§ 62. According to Vogel the attraction is owing to " nervous agency;" or to the immediate action of the exciting cause on the parenchyma, together with, though in some cases only, a change in the blood.

§ 63. Leaving out of view a pre-existing change in the blood, as it in any case can only be accessory not essential to stagnation, it may be asked how does the nervous influence, in comparison with its ordinary and natural operation, act in communicating the attraction for the blood to the parenchyma ? is it by being discharged upon the parenchyma in increased quantity, or by being altogether withdrawn from it ? Again, how does the exciting cause of inflammation, say mechanical or chemical injury, by its immediate action on the parenchyma, call forth an increased attraction in it for the blood ?

§ 64. Without some sort of answer to these questions, the proposition that there is a vital attraction between the blood and parenchyma does not amount to much more than the mere statement of the fact that stagnation takes place in the manner above mentioned.

§ 65. Though he does not attempt any answer to these questions, Emmert goes farther in the way of explanation than Vogel, inasmuch as he points out some of the conditions attending the operation of the attraction. Thus he points out that constriction of the capillaries (small arteries) and attraction between the parenchyma and blood-corpuscles are in antagonism. That when the constriction of the capillaries is *great,* the attraction between the parenchyma and blood is *small,* hence there is no congestion. When, on the contrary, there is *relaxation and dilatation* of the capillaries, there is *great attraction* between the parenchyma and blood : the consequence of which is accumulation and stagnation of red corpuscles.

The indication of these relations is a very important step towards the explanation of the cause of stagnation.

§ 66. It thus appears that the advocates of the attraction-theory, as Henle calls it, have not fully made out their case, in as far as concerns the conditions on which the attraction between the corpuscles and the walls of the vessels, and among the corpuscles themselves depends.

§ 67. Though, as above shown, Henle has not succeeded in giving a satisfactory explanation of the proximate cause of the stagnation of the blood; he has contributed much as regards the theory of inflammation, in tracing how the exciting cause operates in determining the relaxation of the walls of the vessels, with consequent dilatation of their caliber.

§ 68. According to the theory which Henle supports and which he calls neuro-pathological, it is through the nervous system that the exciting cause of inflammation operates, and this, as is also ingeniously argued by Dr. Billing,* by suspending the nervous influence from the small vessels, and consequently determining relaxation of their walls with dilatation of their caliber. To secure a basis for this

* First Principles of Medicine, 4th edition, p. 29. Dr. Billing's explanation of the mode in which congestion is brought about is this: In consequence of exhaustion of the nervous influence, the capillaries become weakened, and allow of over-distension by the ordinary injecting force of the heart, and the part is thus in the state of inflammation or congestion.

theory, Henle enters into a disquisition proving the dependence of the contractility of the vessels on nervous influence. But as this question cannot at present be entered on, reference may be made to Henle's General Anatomy, and to his Report. See also Wagner's Physiology and Billing's Principles of Medicine.

§ 69. Though in answer to the question (§ 33) whether the phenomena of inflammation can be explained by alteration of the vital powers of the vessels in which the blood moves, as narrowed by Dr. Alison down to this—" Does the state of relaxation in which all the vessels are in inflammation afford a sufficient explanation of the changes which take place in inflamed parts?" the same negative conclusion may be come to with Dr. Alison, viz. (§ 52,) that inflammation and its effects are inexplicable by any (mere) alteration of the contractile powers of the living solids concerned in it; still the question may not be so narrowed, and reason may be seen to admit that though mere relaxation does not afford a sufficient explanation of the changes which take place in inflamed parts, the condition on which the relaxation depends may also be the condition of the changes, viz. suspension of nervous influence. Hence, relaxation, though not a cause of, would be coincident with, the changes, and even play a certain part. The relations above referred to (§ 65) as pointed out by Emmert, speak strongly for this coincidence.

§ 70. Preliminary to entering upon an exposition of the theory which appears to the author of this Report to harmonize most completely with all the facts of the case, he postulates the following propositions, delaying what proofs may further be required to support them, in addition to such as have been already adduced or will be adduced further on, until another occasion, when it is proposed to consider the whole subject of contractility and its dependence on nervous influence.

1st. That the constriction and dilatation of the caliber of the small arteries at least, if not of the capillaries, is owing to contraction and relaxation of their walls by virtue of the vital endowment of contractility or tonicity which they possess; the exercise of which contractility is dependent on nervous influence.

2d. That the constant moderate exercise of this endowment on which the ordinary state of tone of the vessels depends, is determined by the constant moderate discharge of nervous influence.

3d. That whilst a greater state of contraction of the vessels than ordinary is owing to an increased discharge of nervous influence, the relaxation, atony, or paralysis of the walls of the vessels on which their dilatation depends, is owing to the suspension of nervous influence.

4th. That the relaxation with dilatation of the vessels from suspension of nervous influence, is the precursor of the retarded flow of blood and stagnation.

§ 71. How the suspension of nervous influence from the walls of the small arteries on which their dilatation depends is produced, involves the question of the mode of operation of the exciting cause of inflammation. To this, as already promised, attention will by and by be directed. At present, inquiry has to be made how the suspension of nervous influence from the small arteries and the consequent relaxation and dilatation of these vessels are connected with the retardation of the flow of blood and subsequent stagnation.

§ 72. In entering upon this inquiry, the author of this Report has, in the first place, to remark that it appears evident that the agglomeration of the red corpuscles of newly abstracted blood is owing to their being withdrawn from some influence under which they were while in the body, an influence which keeps down the tendency to aggregate.

§ 73. The circumstance that the red corpuscles of extravasated blood aggregate shows that that influence is exerted on the blood, not in any part of the body, but only while within the vessels. But the circumstance that the red corpuscles do aggregate in inflammation within the vessels shows that the influence here spoken of may cease to be exerted on the blood even there.

§ 74. Now it has been seen that it is not when the vessels are constricted, and consequently when they are receiving nervous influence, but when they are dilated and when consequently there is a suspension of nervous influence from them that aggregation of the red corpuscles and consequent stagnation of blood takes place in the capillaries. The natural inference from this is that the influence which keeps down the tendency of the red corpuscles to aggregate is communicated to them by the nerves accompanying the small vessels, arteries as well as capillaries, as the blood passes through.

§ 75. When then the nervous influence is withdrawn from the small arteries, and they have in consequence become relaxed and dilated, and when any nervous influence which may naturally be discharged on the capillaries is from the same cause withdrawn, the blood slowly flows through the dilated small arteries into the capillaries as into an indifferent cavity and in the same condition as regards tendency of the red corpuscles to aggregate as blood is when newly drawn from the body, or when extravasated, as well as with the same change in appearance. §§ 5-15-17-60-65.

§ 76. Aggregation of the red corpuscles accordingly takes place, some at the same time adhering to the walls of the vessels. This latter phenomenon is to be attributed in like manner to the suspension of nervous influence from the small vessels. For it is to be observed that the circumstance of the red corpuscles keeping together in the axis of the stream, and aloof from the walls of the vessels in the natural state of the circulation may be accounted for with Mr. Martyn Roberts* by the nervous influence, annulling the attraction of adhesion, or causing a repulsion between the red corpuscles and walls of the vessels at the same time that it does so among the red corpuscles themselves. The suspension of the repulsion between the red corpuscles and walls of the vessels also allows the entrance of red corpuscles in numbers, into the very small vessels into which they before occasionally, and few in number only entered. (§ 10.)

§ 77. The retarded flow of blood which precedes the stagnation, and which, according to Henle, is wholly the physical effect of the dilatation of the paralysed vessels, can be admitted to be so in part only, being greater than the dilatation appears physically to account for. The other cause appears, from what has been above said, to be the commencing attraction among, and agglomeration of the red corpuscles, as also the commencing attraction between them and the walls of vessels. By the dilatation of the vessels, retardation of the flow of blood as a whole, as a fluid is determined; the additional retardation by the commencing attraction, affects the corpuscles only, hence their accumulation in increased quantity while the plasma passes on.†

§ 78. As the retardation of the flow of blood accompanying relaxation and dilatation of the vessels is not alone owing to that dilatation, so on the other hand, the accelerated flow of blood which accompanies constriction of the vessels is not alone owing to that constriction, but in part to diminished attraction, or actual repulsion. The increased discharge of nervous influence which determines the contraction of the walls of the vessels, at the same time calls forth diminished attraction or actual repulsion between the walls of the vessels and the red corpuscles, as well as among the red corpuscles themselves.

§ 79. The view of the process leading to inflammatory congestion which has now been laid before the reader, explains why when, in inflammatory fever, the plasma of the blood in general is much inspissated, and the tendency of the red corpuscles to aggregate when the blood is withdrawn from the body, consequently increased, stagnation does not occur in any set of capillaries in which the flow of blood may be retarded. The nervous influence which continues still to be expended on the small arteries of the part, prevents it by keeping down the tendency of the red corpuscles to aggregate; mere inspissation of the plasma as above shown not being the essential, but merely a promoting condition for the aggregation of the red corpuscles.

§ 80. But of course if in a case in which the plasma is much inspissated, irritation be applied, and relaxation or paralysis and dilatation of the small vessels of

* On the Analogy between the Phenomena of the Electric and Nervous Influences; in the London, Edinburgh, and Dublin Philosophical Magazine for July, 1841.

† Emmert observed that when the crural vein of the frog was tied, the blood, which was in consequence stagnated in the capillaries, presented an equal proportion of plasma and corpuscles.

the part be produced, and consequently the influence keeping down the tendency of the red corpuscles to aggregate be withdrawn, stagnation of the blood will take place with proportionally increased readiness. Inspissated plasma, as above shown, § 50, acting as a promovent of the aggregation, though not itself the essential cause.

§ 81. The view further explains why, when congestion is owing merely to an impediment to the flow of blood in the veins of the part, the red corpuscles do not adhere to each other, and to the walls of the vessels as in true inflammatory congestion.

§ 82. Amidst the obstructed vessels as above mentioned, § 12, a few here and there may still be seen pervious, and through them the mass of blood is directed off in accelerated streams, just as the water of a river would be, if obstructed, by other channels leading from the main channel above the place of obstruction.

The acceleration of the stream of blood is the necessary physical result of the contraction of its aggregate channel.

This indicates that all the nerves of the vessels of the part are not affected. Were all affected there would be mortification of the part. The different forms of inflammation of which a part is susceptible are probably in part owing to a difference in the extent to which the nerves of its vessels are affected.

### V. MODE OF ACTION OF THE EXCITING CAUSE OF INFLAMMATION.

§ 83. That the exciting cause of inflammation acts through the nervous system had been supposed by many, and indeed acknowledged as certain in the case of inflammation of internal organs from cold, but no detailed explanation was attempted of the nature of the part which the nervous system plays until recently. For this pathology is indebted to Henle* and Stilling.†

§ 84. Before going into the subject with Henle and Stilling, it will be proper to notice briefly the opinions of Drs. Macartney, Copland, and Billing.

Granting that increase of blood in a part is an evidence of increased vital power, and vice versâ, it does not follow that the means by which the increase of blood in the part is brought about is increased action of all parts concerned. The action of one part may relax in order to give effect to the action of another. Dr. Macartney not thinking so, and recognizing dilatation of the vessels as a condition of inflammatory congestion, supposed, as above mentioned, that this dilatation of the vessels is a state of activity though one of quite an opposite nature to that of muscle. Instead therefore of a suspension of nervous influence from the small vessels as above admitted, § 68, et seq. Dr. Macartney maintains that there is increased nervous energy. His explanation of how the exciting cause of inflammation operates through the nervous system is in accordance with this, and is consequently quite the opposite of the view to be explained below—in fact so much so, that if read reversed, it would be nearly that to which the reader's attention is about to be directed.

According to Dr. Copland, (who first promulgated his views on inflammation many years ago,) in sthenic inflammations, organic nervous influence, and vascular action, are not only primarily increased, but also otherwise changed. Dr. Copland's views regarding asthenic inflammation, are more in accordance with what is maintained in this Report, as to inflammation generally.

Dr. Billing, as above shown, (§ 68,) maintains the view that inflammation is primarily owing to exhaustion of the nervous influence, which gives the capillaries power. This exhaustion is produced by continued excitation of the nerves.

§ 85. Though explaining differently its mode of action, the author of this Report has above recognized with Billing and Henle, as the essential condition of stagnation of the blood in inflammation, suspension of nervous influence from the small vessels with consequent relaxation of their coats, and dilatation of their caliber.

* Pathologische Untersuchungen, 1840, and also 'Bericht,' ut supra.

† Physiologische, pathologische und medicinisch-praktische Untersuchungen über die Spinal-irritation, 1840.

An inquiry how the exciting cause of inflammation operates in producing this suspension of nervous influence now claims attention.

§ 86. The theory which Henle, by his physiological investigation of the subject, has been led to form of the mode of action of the exciting cause of inflammation, in determining the suspension of nervous influence from the small vessels on which their relaxation and dilatation depend, is this:

The exciting cause, of what nature soever it may be, whether external or internal, acts primarily on sensitive nerves, exalting their activity. The motor nerves of the vessels which have sympathetical relations with the excited sensitive nerves, are secondarily affected. But this affection of the motor nerves of the vessels, which supervenes by reflex action on the excitement of the sensitive nerve, is not a corresponding state of excitement, but an opposite one of depression, of suspension of action, of paralysis.

§ 87. This form of sympathy, in which the state of excitement of one nerve determines depression of another, Henle calls *antagonism;* the name of *sympathy* in a restricted sense being retained for that form in which a state of activity of one nerve is called forth by a corresponding state of another. This latter form is more common in the domain of the cerebro-spinal system; the former in the domain of the ganglionic system, the source of the nerves of the vessels.

§ 88. Sometimes, however, sympathy is exemplified in the vessels by constriction supervening on irritation and preceding dilatation. But in most cases relaxation and dilatation of the vessels from suspension of nervous influences, are the primary effect of the irritation, no matter whether that irritation have been violent or moderate. Hence Henle contends that the relaxation of the vessels on which their dilatation depends cannot be a mere consequence of exhaustion of the vessels from previous action, as has been suggested by Alison (p. 117) and Billing, but can only be antagonistic. Into this, however, it is not necessary to enter; for, provided suspension of nervous influence and consequent dilatation of the vessels, do take place, it is indifferent for the theory of the proximate cause of inflammation above expounded whether that state of the vessels be the result of antagonism or of exhaustion succeeding a state of activity induced by sympathy.

§ 89. Inflammation excited by exposure to cold often affects some part other than that to which the cold was immediately applied. In such a case it may be said *hic stimulus, ibi fluxus*, but in most cases of external, traumatic inflammation which come under notice, the congestion occurs at the place where the irritation was applied, *ubi stimulus, ibi fluxus.* Hence the widely-spread belief that the irritation affects the vessels directly; but to say nothing of physiological examples of reflexion on remote vessels, *hic stimulus, ibi fluxus,* which may be adduced in contradiction of the belief referred to, such for example as the circumstance, that irritation of the conjunctiva, or of the mucous membrane of the nose, excites the congestion in the lacrymal gland on which the discharge of tears, resulting from the irritation depends, a pathological one, in various ways more instructive, will be adduced below in the inflammatory congestion of the conjunctiva and sclerotica which supervenes on a wound of the cornea.

### VI. EXPLANATION OF THE OCCURRENCE OF INFLAMMATION OF A PART AFTER SECTION OR DISEASE OF ITS NERVES.

§ 90. In those cases in which inflammation of an organ occurs after section of some part of the sympathetic system—of the eye for example after section of the sympathetic in the neck, as also in those cases in which inflammation of the eye supervened, on section of the fifth pair, and of the lungs and stomach on section of the par vagum—the inflammation was at first attributed to the suspension of some peculiar influence supposed to be exerted by the nerves over the nutritive processes. Dr. Alison, however, combated the opinion that the nutritive processes are in any direct manner under nervous influence. And in regard to the cases in which inflammation of the eye, lungs, and stomach supervened on section of their nerves, supposing that these nerves are wholly sensitive, he suggested that the inflammation, instead of being a direct effect of their section, might rather be an indirect result of the suspension of sensation produced by the section; and in this way: He supposed that due secretion on the surface of the mucous membranes implicated is determined by the exigencies of the part in this respect being made known as it were by the sensitive nerves. These nerves being cut, the secretion becomes diminished and altered, the consequence of which is that irritation by foreign matters is allowed to operate to an extent to excite inflammation. Dr. Alison does not offer any explanation of the occurrence of inflammation of an organ after section of some part of the sympathetic.

§ 91. The progress of physiology has confirmed Dr. Alison's opinion as to the immediate non-dependence of the nutritive processes on the nervous system. Nerves act indirectly only, and that by virtue simply of their ordinary sensiferous and motiferous endowments. Even the sympathetic as first declared by Stilling, acts in no other way. And why it appears to be more particularly the nerve governing nutrition is explained by the circumstance that it is the principal source of the nerves of vessels.

§ 92. In regard to inflammation of an organ occurring after section of some part of the sympathetic, Stilling declares it to be owing simply to paralysis of the walls of the vessels, from section of the source whence their motor nerves are derived. And this, taken in conjunction with the theory of the proximate cause of inflammation above enunciated, appears to be the true and natural explanation.

§ 93. The inflammation of the eye after section of the fifth pair, and of the lungs and stomach after section of the par vagum, Stilling declares to be the effect of paralysis also, but determined in the following indirect manner:—He supposes that a reflex action from sensitive nerves to the nerves of vessels is constantly going on, and is a necessary condition of the activity of the vessels. When, therefore, the sensitive nerves are cut, a suspension of this reflex action takes place; the consequence of which is paralysis of the nerves of the vessels.

§ 94. Henle objects to this view of Stilling,—and the objection is also applicable to Alison's,—that were the integrity of sensitive nerves a *conditio sine qua non* for the normal function of the vessels, the loss of sensibility must in every case be followed by stagnation of the circulation; which is not the case, for there are anæsthesiæ in which the circulation in the part goes on; when the nerves of the leg of the frog are cut, for example, the circulation nevertheless continues.

§ 95. According to Henle, the stagnation of blood which takes place after section of sensitive nerves, the fifth pair, the par vagum, &c., belongs to a category with those which occur after section of branches of the sympathetic. It must only be granted that the sympathetic or nerves of vessels are mixed with those so-called sensitive nerves. There are in favour of this, not only anatomical facts, such as the passing of branches from the spheno-palatine ganglia to the twigs of the trigeminus, but also physiological analogies; viz., the collection of other motor nerves, e.g. for the pharynx, gullet, and respiratory organs, in the trunk of the *vagus*. In paralysis of these nerves, from affection of their central ends, the nerves of the vessels are not necessarily implicated, and therefore remain active;* in the section of the nerves of the extremities, in Hausmann's cases, they were not injured, because they accompany the vessels.

§ 96. In like manner, the continuance of the circulation in the frog's leg after section of its nervous trunks is, perhaps, owing to the nerves of the vessels not being included in the trunks, and therefore not implicated in the section. But when the source of these nerves is implicated, the circulation in the small vessels is affected, as shown by the experiments of Flourens and Baumgärtner, in which injury of the spinal cord affected the flow of blood in the capillaries.

§ 97. According to Magendie, the inflammatory symptoms in the eye are more violent after section of the first branch of the fifth than after the section of the trunk above the ganglion of Gasser. This fact, which is inexplicable according to Stilling's view, is, Henle thinks, easily intelligible according to his;—for in the first

* See also Macartney on Inflammation, p. 133.

case, all the nerves of the vessels, including those contributed by the sympathetic, are cut; in the second case, probably only a small number, which are from the first mingled with the trigeminus.

§ 98. The readiness with which inflammation may be excited by slight irritation in paralysed limbs, may be collated with the readiness with which reflex action occurs in the same limbs. As slight irritation calls forth by reflexion contraction of the muscles (sympathy), so a similarly slight irritation calls forth by reflexion relaxation of the vessels (antagonism.)

§ 99. The inflammation which sometimes accompanies spinal irritation, or neuralgia, Stilling explains in the same way as he does that which results from the section of the fifth pair for instance, he supposing that in neuralgia the sensitive nerves are in a state of paralysis. Henle maintains the opposite, and explains the inflammation on the principle of antagonism above mentioned, thus:—Neuralgia being a state of excitement of a sensitive nerve, determines antagonistic paralysis of the motor nerves of the vessels of the part, whence relaxation of their walls and dilatation of their caliber.

§ 100. In the preceding part of this Report, where the immediate cause of the stagnation of the blood in inflammation is under discussion, no reference is made to Stilling's views on the subject. Here it may be mentioned, that in addition to the set of cases above considered; viz., 1st, those occurring after section of the sympathetic; 2d, those occurring after section of sensitive nerves; and 3d, those accompanying spinal irritation; in which he says that there is paralysis of the nerves and relaxation and dilatation of the vessels; he admits another set of cases—traumatic —in which, on the contrary, there is constriction of the vessels in consequence of increased action of their motor nerves, determined from reflexion, from increased excitement of the sensitive nerves. He does not, however, attempt any detailed explanation of how the paralysis of the nerves and relaxation and dilatation of the vessels, in the one case, or the excitement of the nerves and constriction of the vessels, in the other, determine the stagnation. In the latter case, in particular, he makes no use of the alleged increased tone of the vessels in giving an explanation of the cause of the stagnation of the blood, but ascribes it to a morbid condition of the blood; thus, as Henle remarks, giving up his own principle, that the cause of inflammation operates through the nervous system, and calling into his aid a humoral pathological theory exactly in cases in which a change of the humours is least probable.

### VII. EXUDATION.

§ 101. Immediately after or during the stagnation of the blood, exudation commences. From being at first serous the exuded fluid comes at last to be pure plasma, or at least a fluid containing a greater or less quantity of fibrin.

§ 102. The exudation may, in the aggregate, be attributed to the thinning of the walls of the vessels, from their relaxation and dilatation on the one hand, and the pressure from within the vessels on the other. Besides these, another condition suggests itself as likely to promote exudation, viz., the circumstance that the plasma will be pressed out from among the aggregating corpuscles, even when the blood would not, if out of the body, present the buffy coat, and that because within the body the fibrin of the plasma does not so readily coagulate.

§ 103. When, however, the mass of blood has already become changed to that condition in which the buffy coat would present itself were the blood drawn from the body, the plasma, at the same time that it is more quickly and energetically squeezed out from among the aggregating red corpuscles, will present itself in greater quantity and richer in fibrin, for transudation through the walls of the capillaries.

§ 104. The question, however, occurs, why does serum alone pass out first? The author of this Report thinks, with Dr. Watson, (Lectures on the Practice of Physic, vol. i, p. 155,) that it is, as in common œdema, owing to obstruction; the obstruction in inflammation being from the stagnation of the blood. But how obstruction determines exudation of serum alone, remains a question. To help to a solution of this, it may be stated that, according to Kürschner, water passes most quickly through animal membranes, and saline solutions more quickly than viscid, gummy, and albuminous solutions.

§ 105. None of the corpuscles of the blood pass out along with the exuded fluid as long as the vessels are entire. But it is often observed that at certain points the walls of the vessels in which the blood was stagnated, have given way, and permitted an extravasation of both red and colourless corpuscles.

§ 106. With exudation is completed the inflammatory process, properly so called.

### VIII. INFLAMMATION OF NON-VASCULAR PARTS.

§ 107. In certain non-vascular parts morbid actions may go on in all respects similar to those which usually attend or result from inflammation. The cornea, for example, though it is vascular whilst being developed, is, in its fully formed and healthy state, non-vascular; and yet inflammation of the cornea is spoken of.

§ 108. The cornea, there is reason to believe, derives the materials necessary for its nutrition from the blood circulating in the vessels of the adjoining parts of the conjunctiva and sclerotica. Let it be inquired what takes place in the cornea when there is applied to it such an irritation as would excite inflammation in one of the vascular parts of the eye.

§ 109. When the cornea is injured then, congestion of the vessels, of the adjoining parts of the conjunctiva and sclerotica takes place, and exudation into the substance of the cornea by and by ensues. Thus, though non-vascular, and of course not the seat of inflammatory congestion, it becomes the seat of a very important part of the inflammatory process—the most important part, perhaps, as regards the events of the process.

§ 110. The cornea in this state may therefore be said to be, to all intents and purposes, inflamed—the only difference in respect to it, as compared with vascular parts, being that the vascular congestion is *not in it*, but *in adjoining structures*.

§ 111. On the other hand, it is to be remarked, that although these adjoining structures are the seat of the congestion, little or no exudation may take place in them or on them, and they may therefore be said scarcely or not at all to be the seat of inflammation as regards the events of the process. When the conjunctiva and sclerotica are really inflamed, exudation in or on them may occur; but then the congestion is different in seat and extent from what it is in the former case, and there may be no exudation into the cornea—the cornea may remain unaffected.

§ 112. In the progress of inflammation of the cornea, this structure may become vascular, but such an event is owing to the development of new vessels, such as happens in inflammation of vascular parts, and as will be considered on another occasion.

§ 113. Though inflammation of the cornea considered as a non-vascular part, has been thus dwelt on, the truth is that all tissues as regards their component elements are properly speaking non-vascular, and differ from the cornea, only in degree of proximity to the vessels, and therefore in inflammation only in degree of the proximity to the source of the exudation.

§ 114. But this very difference presents a natural analysis of the inflammatory process. It enables one to observe separately, the two great stages of inflammation proper, the *congestion* and the *exudation*,—the congestion in one place, the exudation in another. It also enables one to observe, as will be shown on another occasion, in an uncomplicated manner, the eventual stages of inflammation, such as reorganization and suppuration. Lastly and especially, it enables one to analyse the mode in which the inflammatory irritation is communicated to the vessels, in other words, the mode of action of the exciting cause.

§ 115. In what is ordinarily called a vascular part, the irritation, for aught that could be said to the contrary, except by a round about process of reasoning as above seen, might act directly on the vessels as some maintain, but in the case of irritation applied to the cornea alone, and not either to the conjunctiva or sclerotica, it cannot do so. And for the very simple reason that there are no vessels in it to be acted on. The vessels which are affected are those of the conjunctiva and sclerotica.

§ 116. The mode in which these vessels are affected in consequence of irritation applied to the cornea alone appears to be this: Excitement of the sensitive nerves of the cornea, (for the cornea has nerves, though no vessels, as at first shown by Schlemm, and since found by Valentin, Pappenheim, and others,) calls forth antagonistically, according to Henle's principle, a state of depression, a temporary paralysis of the motor nerves of the contractile fibres of the walls of the small arteries opening into the capillary network of the conjunctiva and sclerotica, adjoining the cornea. The consequence of this is first relaxation and dilatation of those arteries, and then accumulation and stagnation of blood in the capillaries in the manner already explained.

In the foregoing Report, no reference has been made to Mr. Travers's recent work on Inflammation, as it is principally occupied with the events of inflammation and the healing process. There is indeed nothing in it on *the theory of inflammation proper*, beyond the statement of the fact of stagnation of the blood in the vessels, and the effusion or exudation contingent on it.

---

